# Supplementary figures and images for: New isolates refine the ecophysiology of the Roseobacter CHAB-I-5 lineage
Source: ISME Commun. 2025 Apr 18;5(1):ycaf068. doi: 10.1093/ismeco/ycaf068 (PMC12075776; doi:10.1093/ismeco/ycaf068)

Other Alphaproteobacteria

Other Roseobacter

CHAB-I-5

Other Alphaproteobacteria

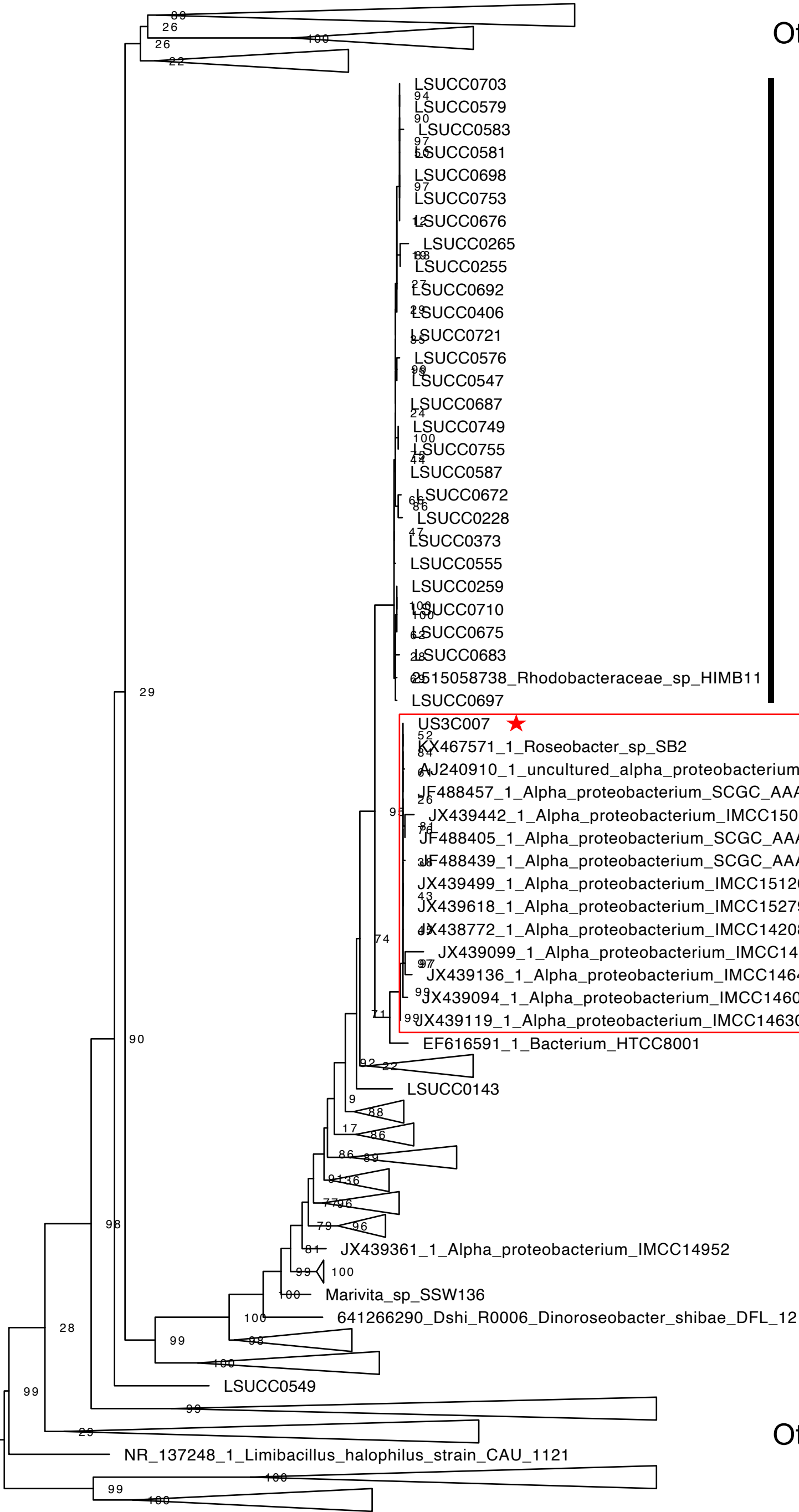

0.08

Supplement: FigS1_16S_phylogeny_collapsed_nodes_121920_AFDpdf_ycaf068 [file figs1_16s_phylogeny_collapsed_nodes_121920_afdpdf_ycaf068.pdf]

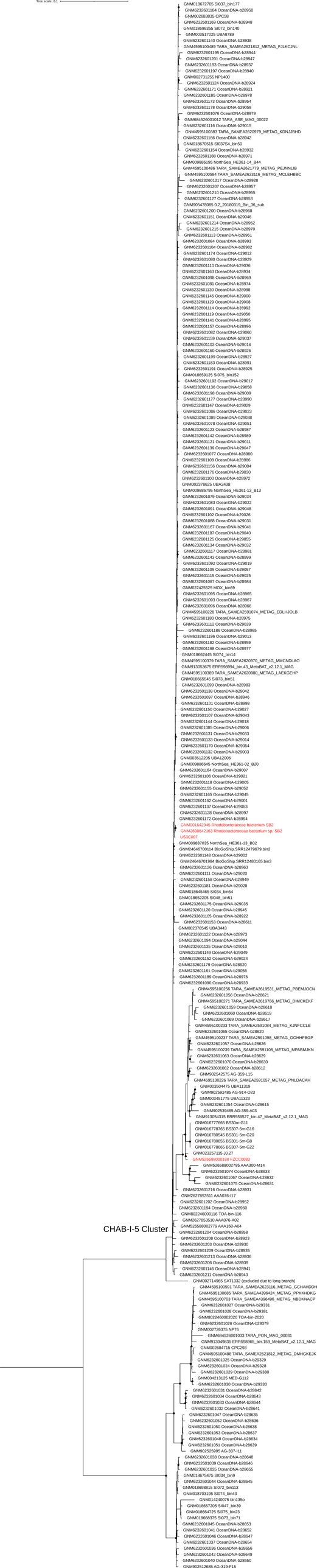

Supplement: FigS2new_ycaf068 [file figs2new_ycaf068.pdf]

Tree scale: 0.01

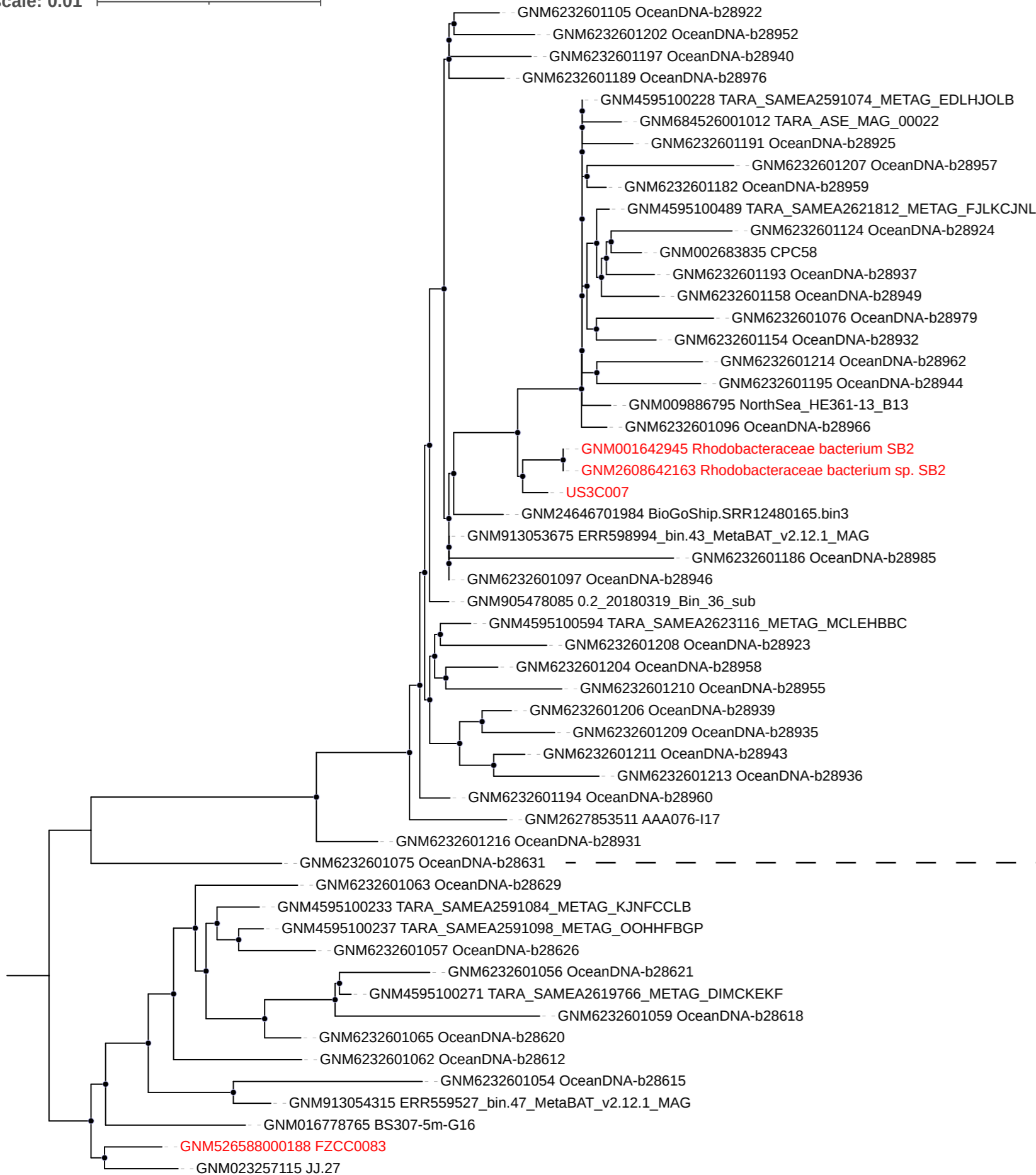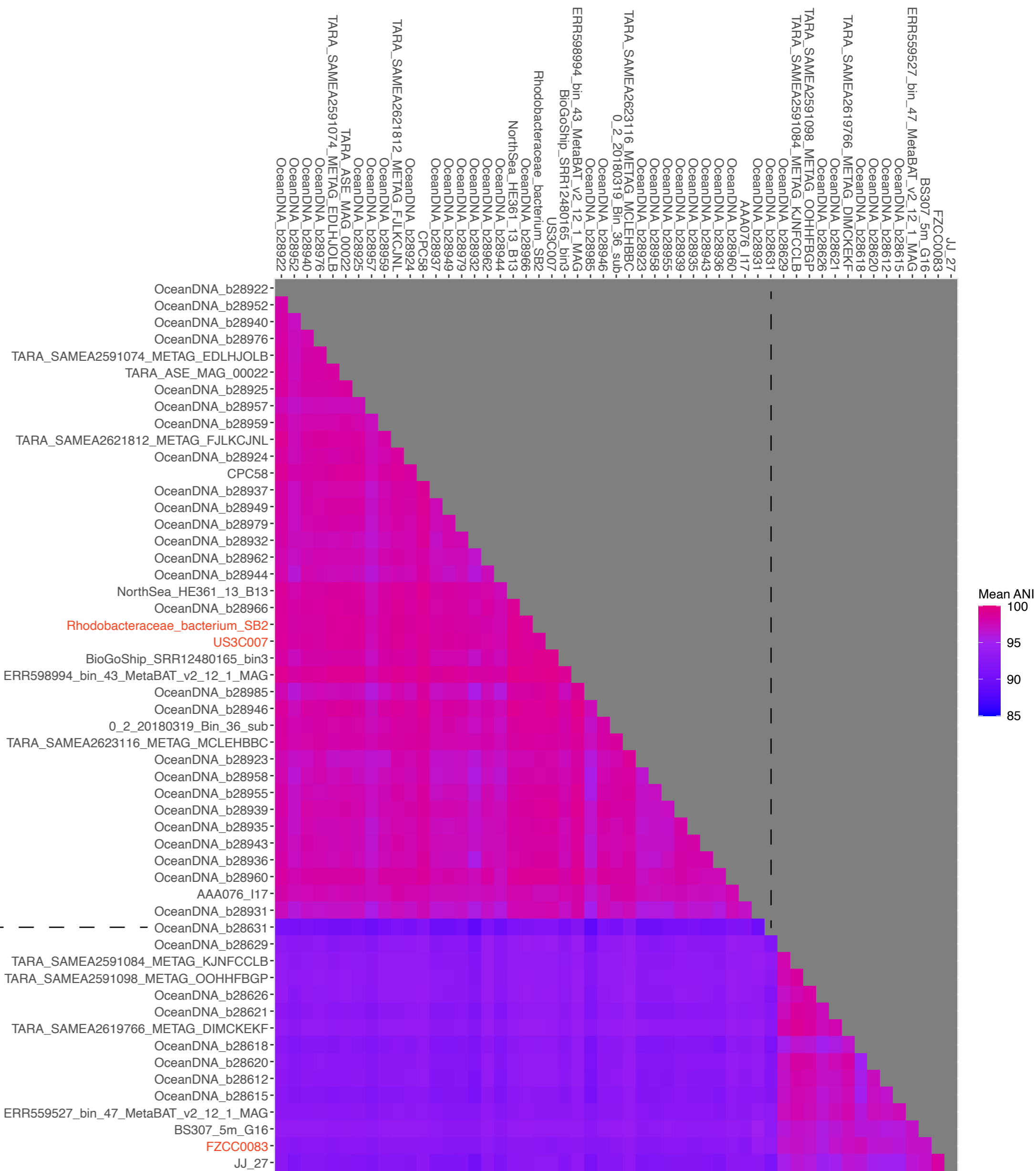

Supplement: FigS3_prune54_ANI_ycaf068 [file figs3_prune54_ani_ycaf068.pdf]

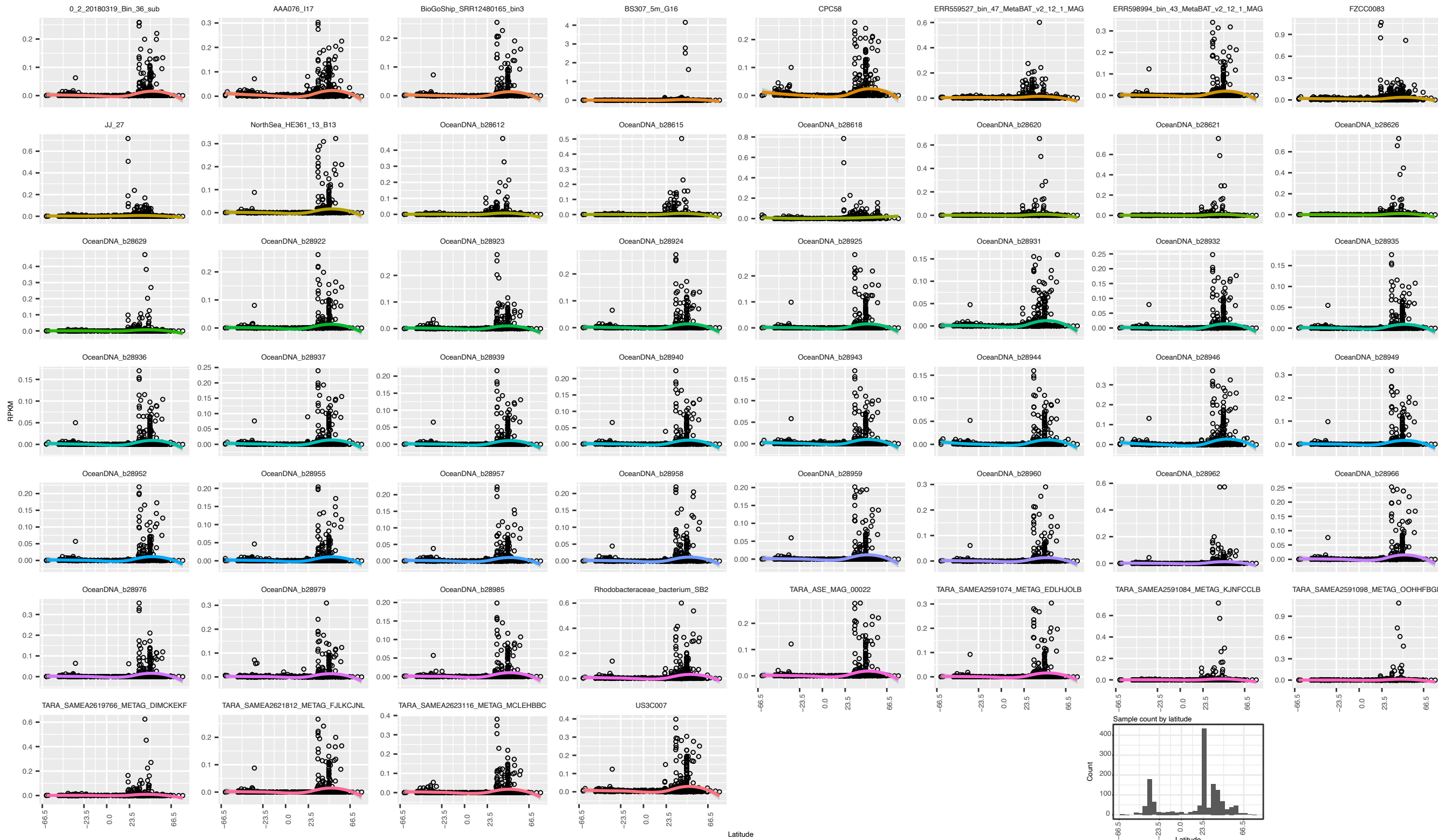

Supplement: FigS4_052024_ycaf068 [file figs4_052024_ycaf068.pdf]

A

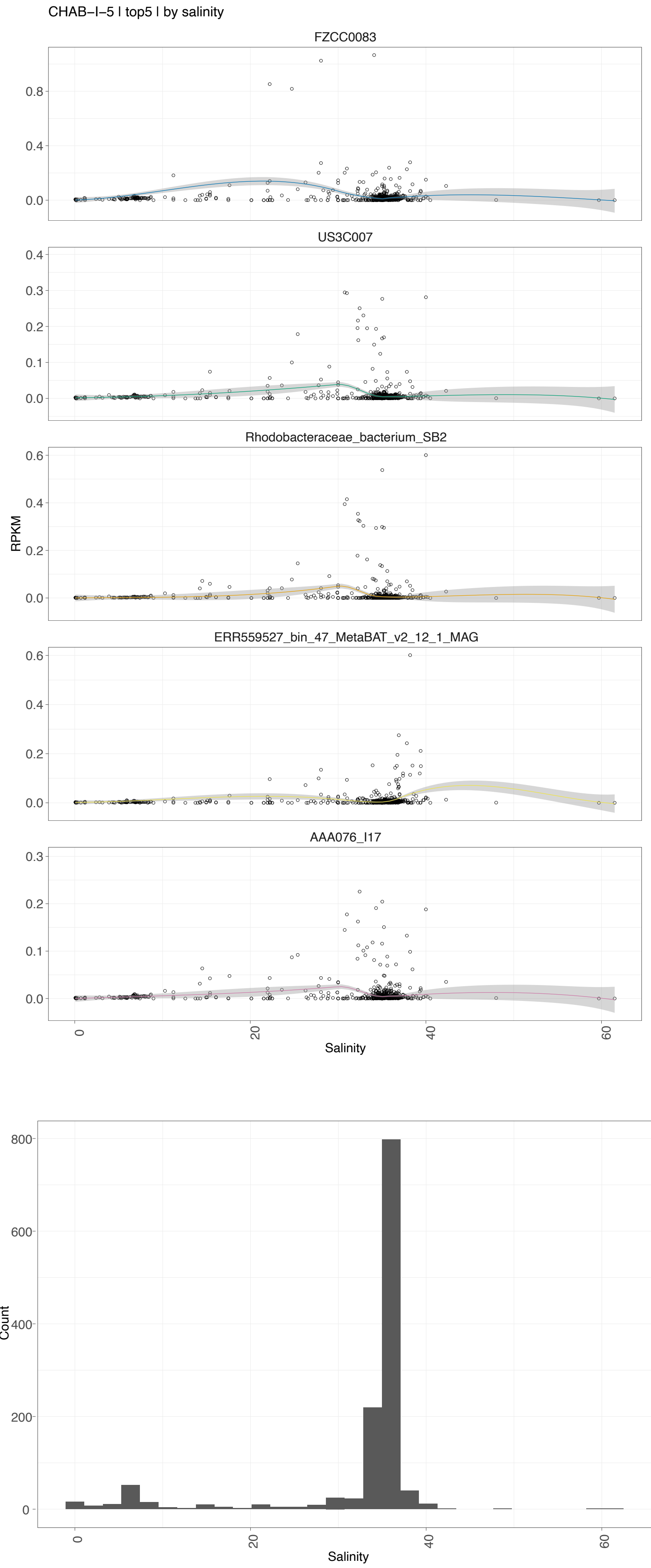

B

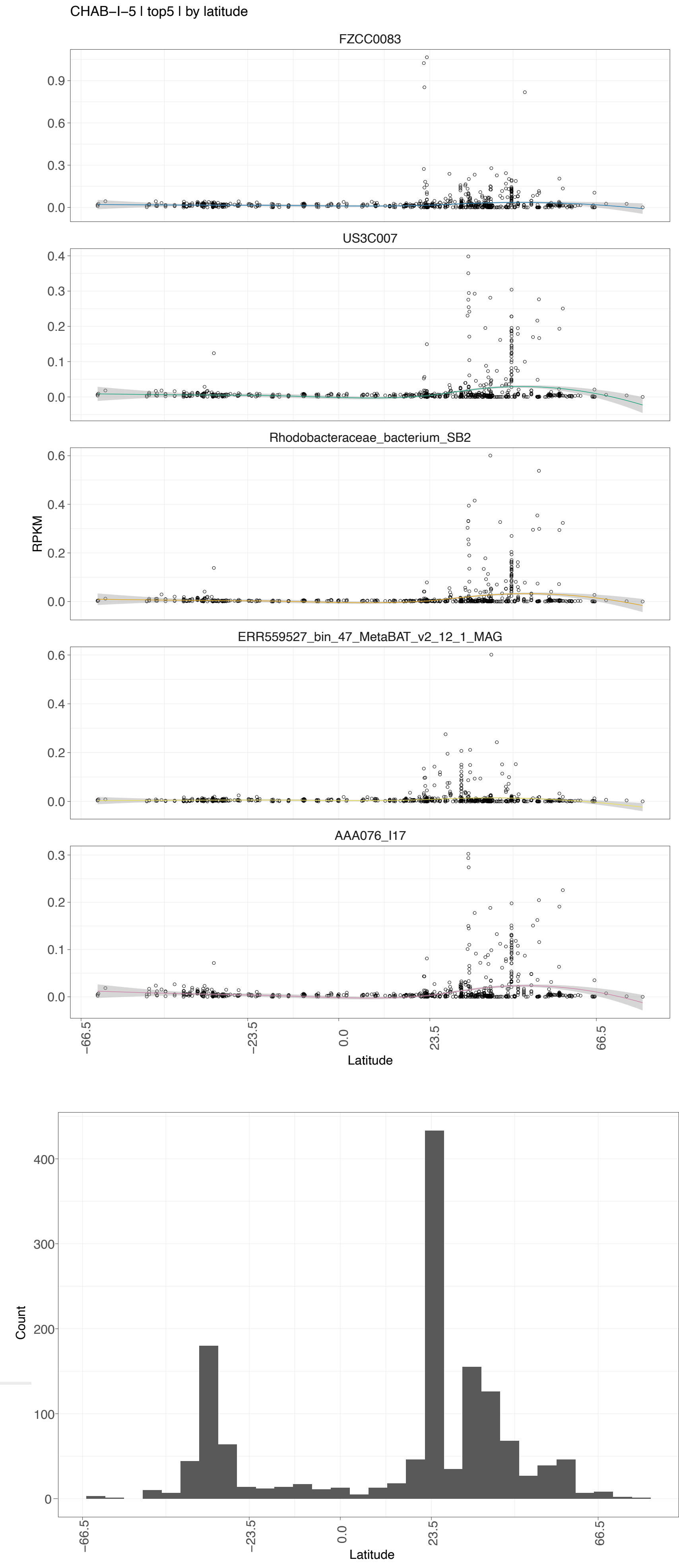

C

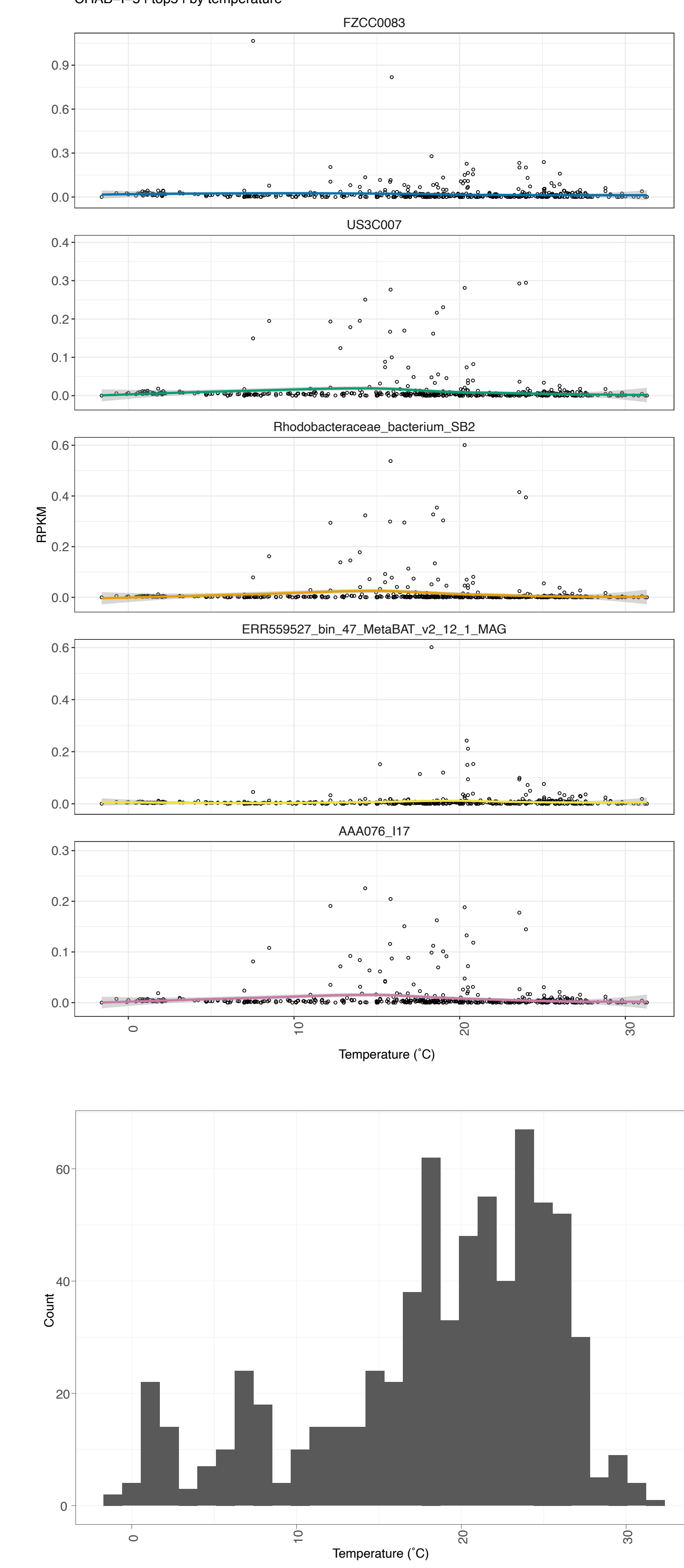

Supplement: FigS5_052024_ycaf068 [file figs5_052024_ycaf068.pdf]

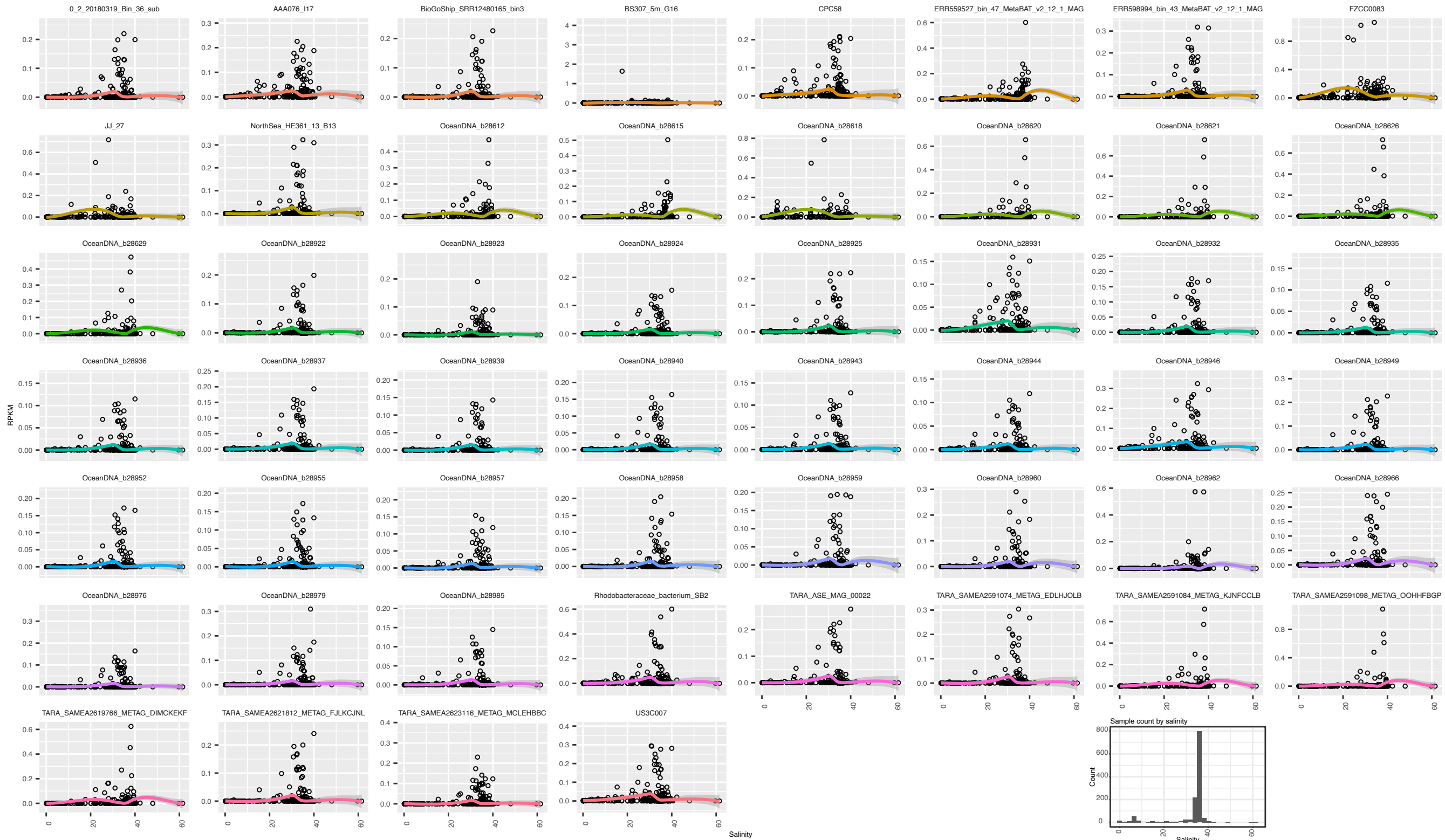

Supplement: FigS6_052024_ycaf068 [file figs6_052024_ycaf068.pdf]

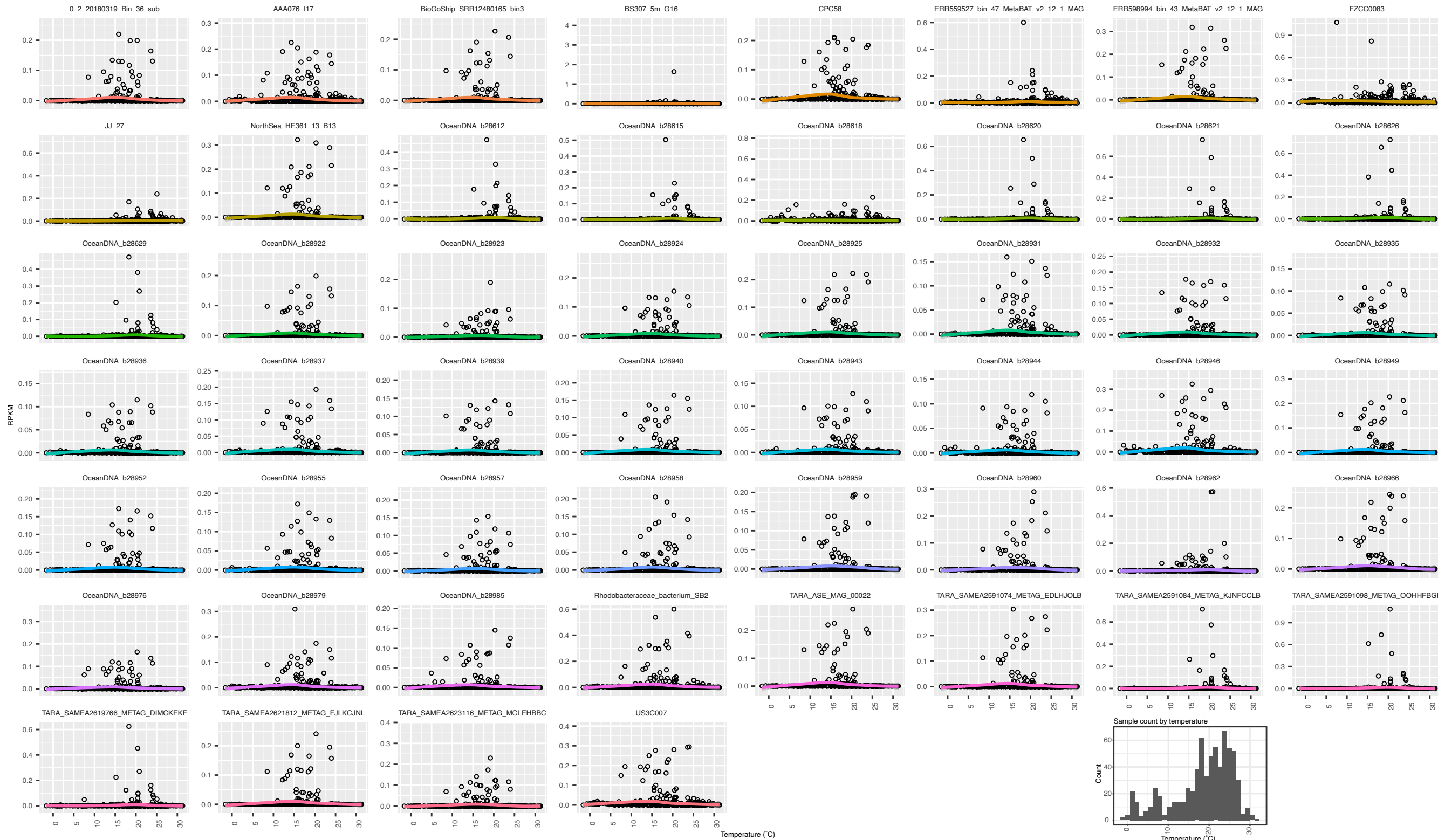

Supplement: FigS7_052024_ycaf068 [file figs7_052024_ycaf068.pdf]

# Salinity

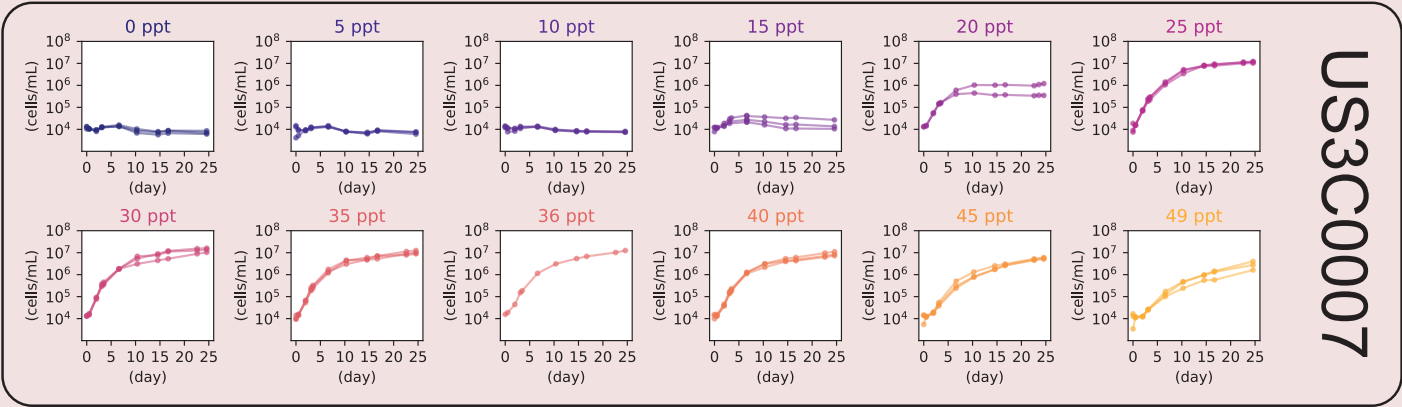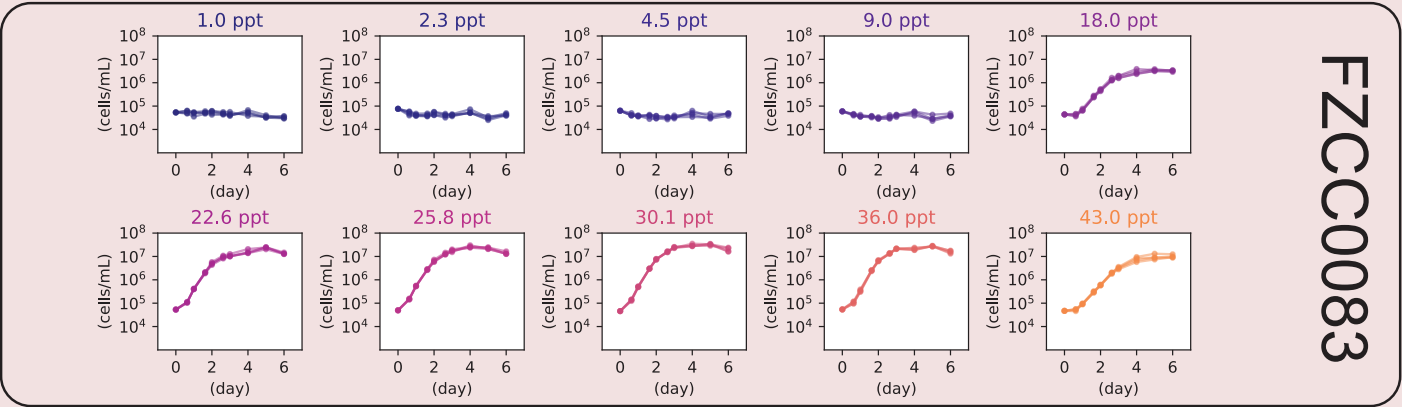

# Temperature

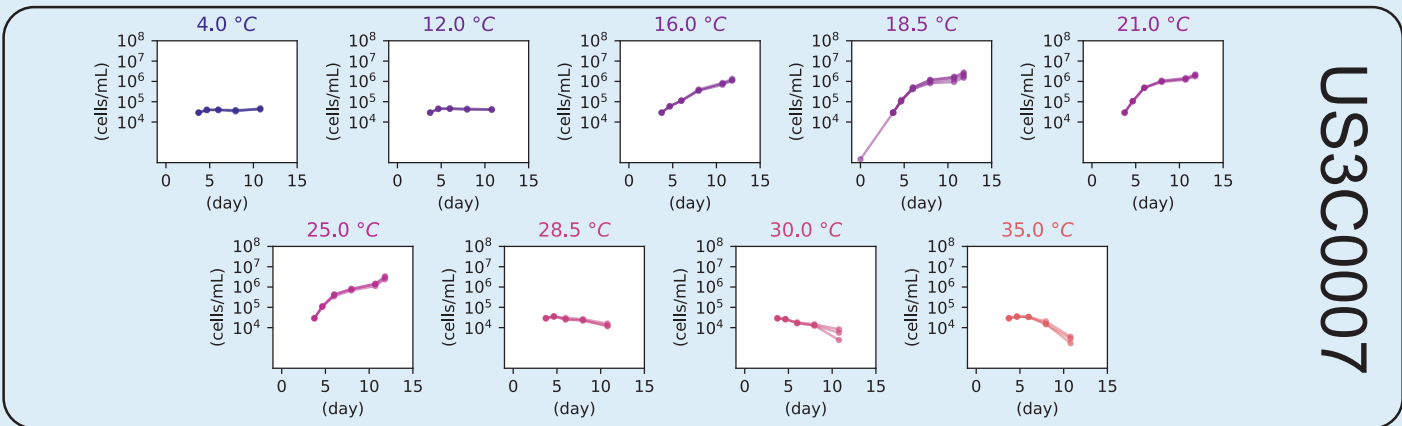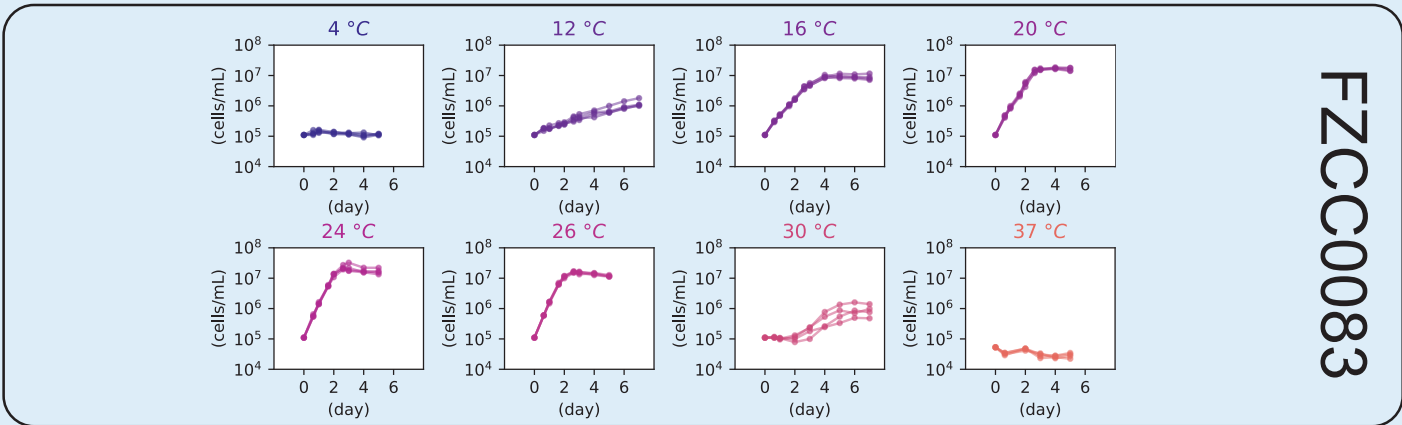

Supplement: FigS8_all_growth_curves_ycaf068 [file figs8_all_growth_curves_ycaf068.pdf]

# CHAB-I-5 summed clusters by temp-linear

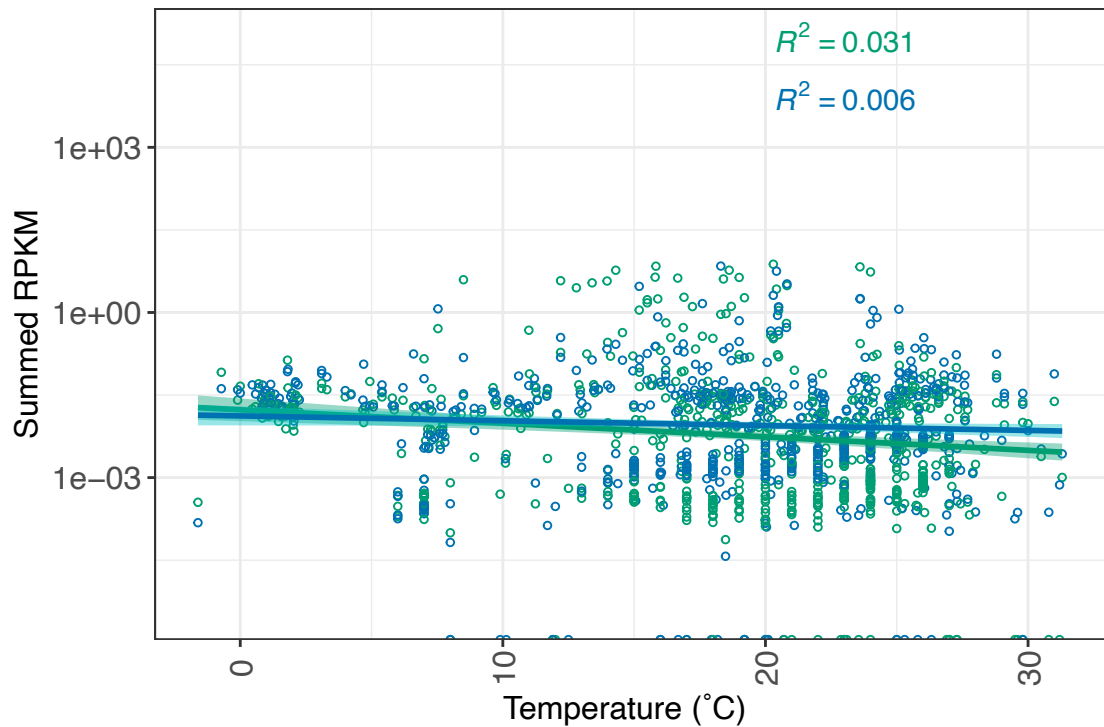

Supplement: FigS11_052024_ycaf068 [file figs11_052024_ycaf068.pdf]
