# Supplementary material for: New isolates refine the ecophysiology of the Roseobacter CHAB-I-5 lineage
Source: ISME Commun. 2025 Apr 18;5(1):ycaf068. doi: 10.1093/ismeco/ycaf068 (PMC12075776; doi:10.1093/ismeco/ycaf068)

$V = \frac{4}{3}\pi r^3 + \pi r^2 h$

$S = \pi r^2 + 2rh$

$h = (S - \pi r^2)/2r$

$l = h + 2r$

$V = \frac{4}{3}\pi r^3 + \pi r^2 \cdot (S - \pi r^2)/2r$

$= \frac{4}{3}\pi r^3 + \pi r \cdot (S - \pi r^2)$

$= \frac{4}{3}\pi r^3 + S \cdot \pi r - \pi \cdot \pi r^3$

$= (\frac{4}{3} - \pi)\pi r^3 + S \cdot \pi r$

$SA = 2\pi r(2r + h)$

$= 2\pi r(2r + (S - \pi r^2)/2r)$

$= 4\pi r^2 + \frac{2\pi r(S - \pi r^2)}{2r}$

$= 4\pi r^2 + \pi S - \pi^2 r^2$

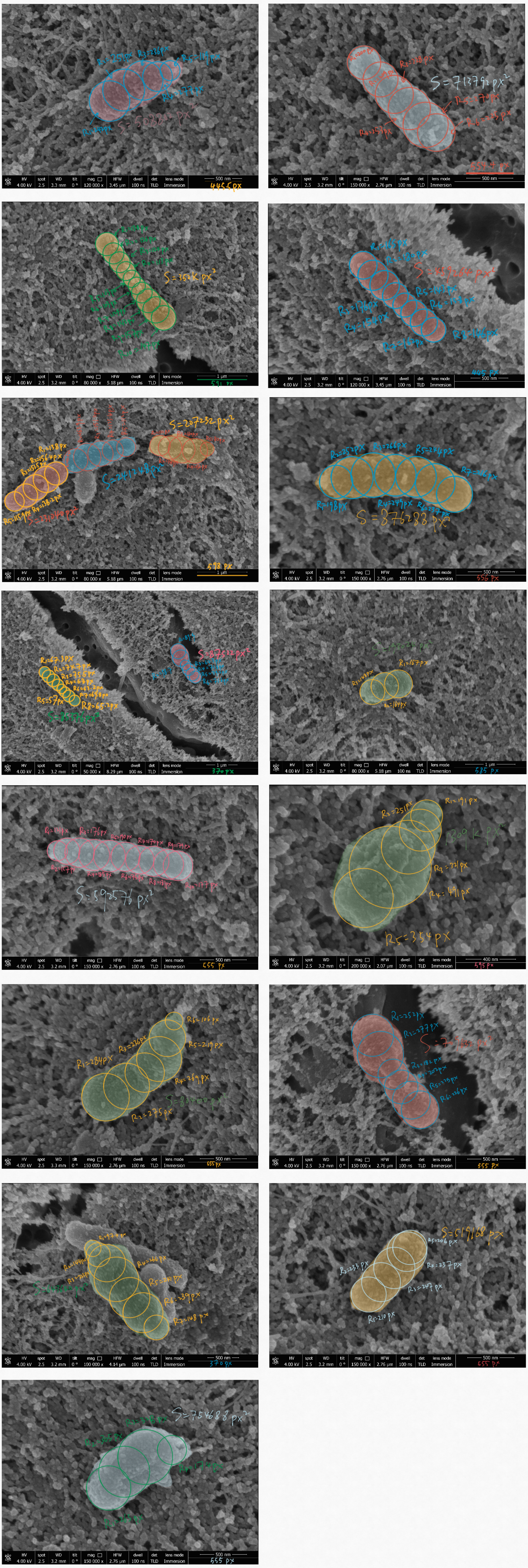

Supplement: FigS9_US3C0007_analysis1_ycaf068 [file figs9_us3c0007_analysis1_ycaf068.pdf]
