# Supplementary material for: New isolates refine the ecophysiology of the Roseobacter CHAB-I-5 lineage
Source: ISME Commun. 2025 Apr 18;5(1):ycaf068. doi: 10.1093/ismeco/ycaf068 (PMC12075776; doi:10.1093/ismeco/ycaf068)
Supplement: FigS10_US3C0007_analysis2_ycaf068 [file figs10_us3c0007_analysis2_ycaf068.pdf]

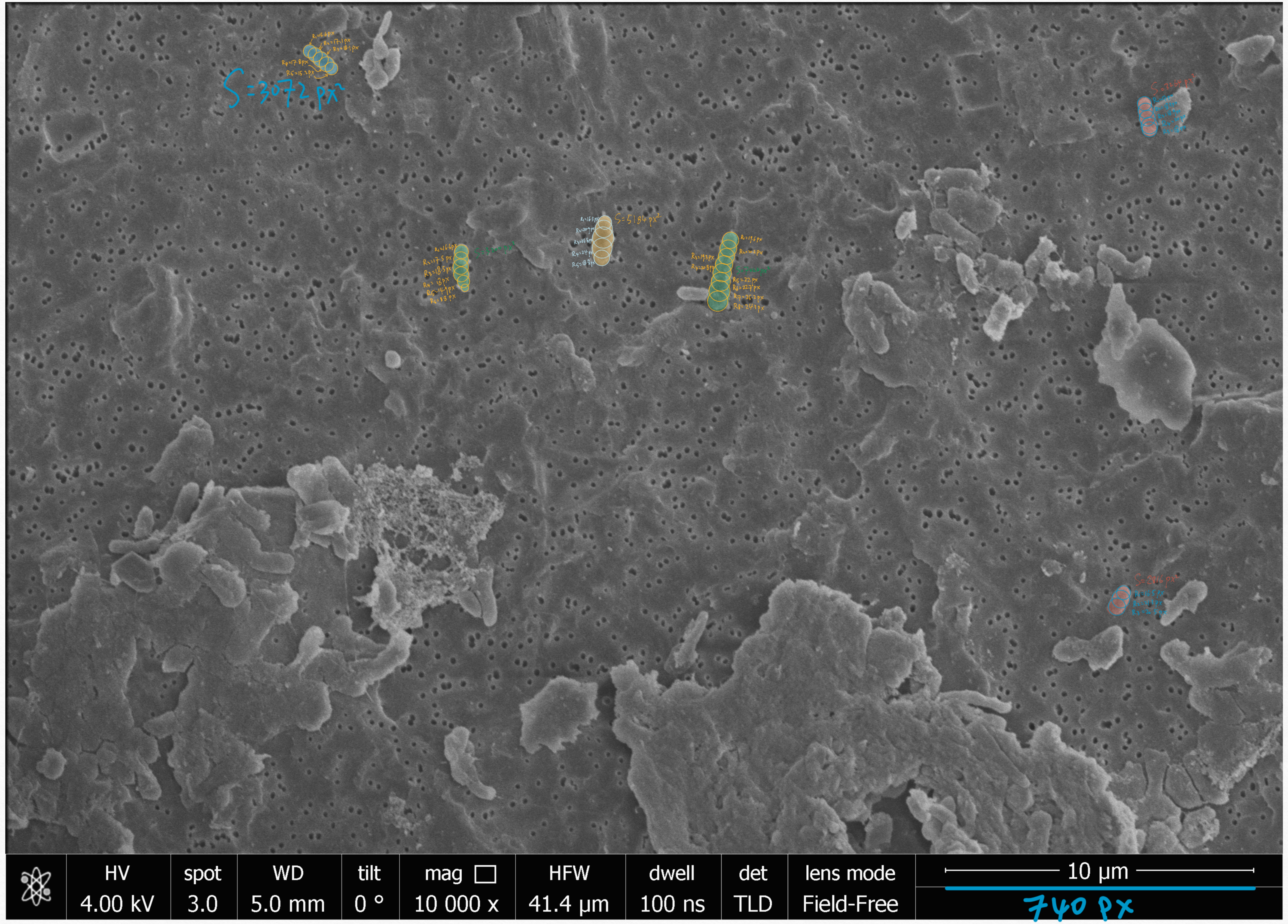
S=3072 px²
HV 4.00 kV
spot 3.0
WD 5.0 mm
tilt 0 °
mag 10 000 x
HFW 41.4 µm
dwell 100 ns
det TLD
lens mode Field-Free
10 µm
740 px

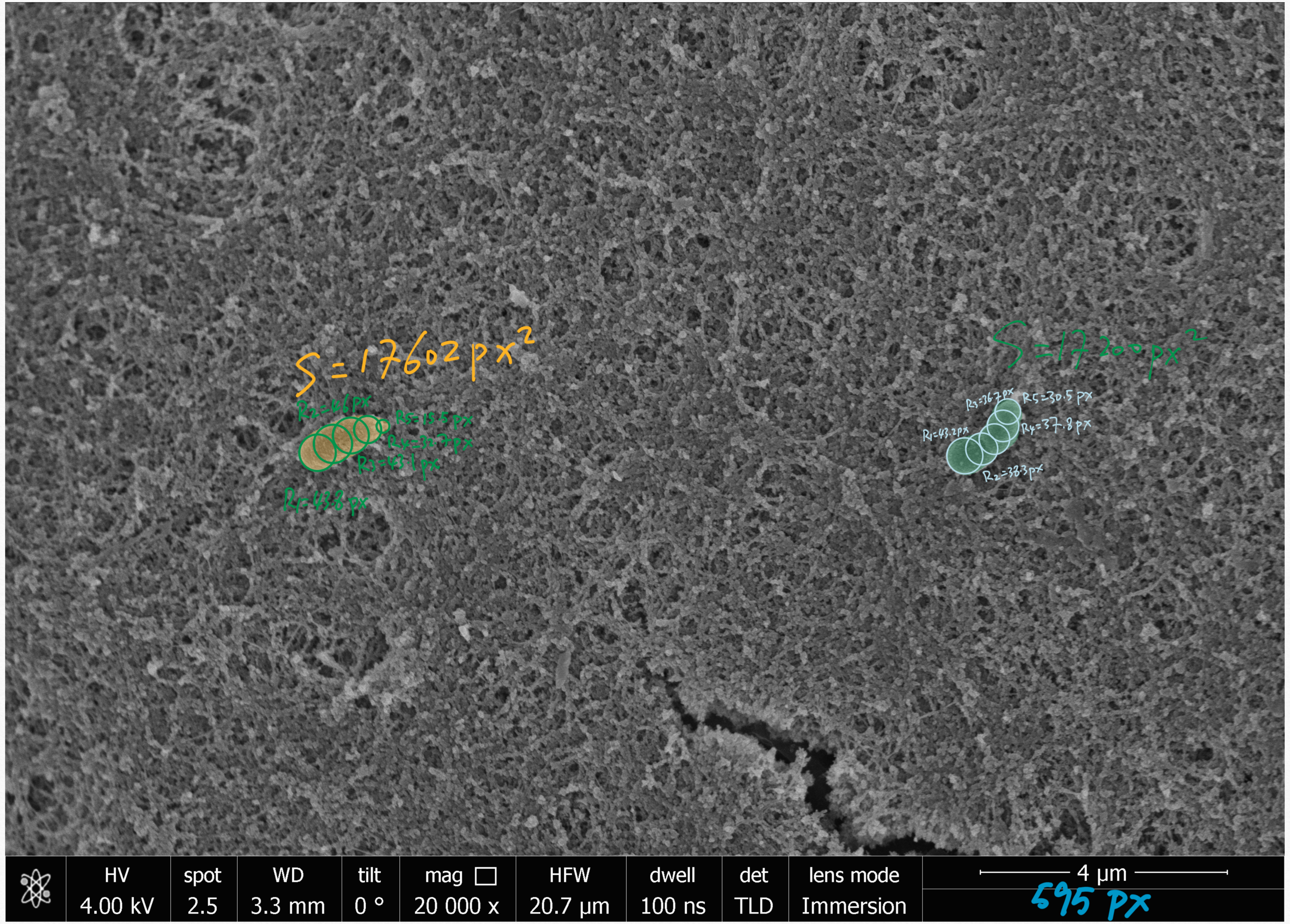
S=17602 px²
HV 4.00 kV
spot 2.5
WD 3.3 mm
tilt 0 °
mag 20 000 x
HFW 20.7 µm
dwell 100 ns
det TLD
lens mode Immersion
4 µm
595 px
