# Supplementary material for: New isolates refine the ecophysiology of the Roseobacter CHAB-I-5 lineage
Source: ISME Commun. 2025 Apr 18;5(1):ycaf068. doi: 10.1093/ismeco/ycaf068 (PMC12075776; doi:10.1093/ismeco/ycaf068)
Supplement: FigS12_coverage_ycaf068 [file figs12_coverage_ycaf068.pdf]

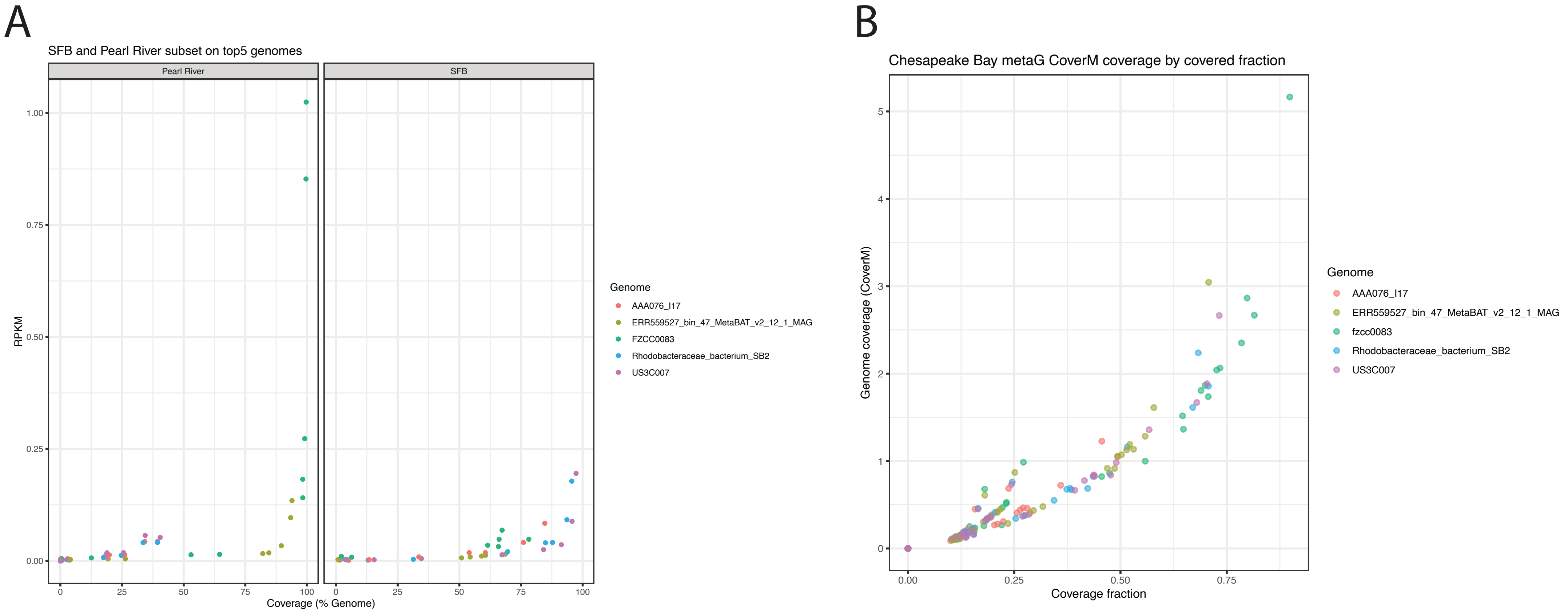
A
SFB and Pearl River subset on top5 genomes
Pearl River
SFB
RPKM
Coverage (% Genome)
Genome
AAA076_I17
ERR559527_bin_47_MetaBAT_v2_12_1_MAG
FZCC0083
Rhodobacteraceae_bacterium_SB2
US3C007
B
Chesapeake Bay metaG CoverM coverage by covered fraction
Genome coverage (CoverM)
Coverage fraction
Genome
AAA076_I17
ERR559527_bin_47_MetaBAT_v2_12_1_MAG
fzcc0083
Rhodobacteraceae_bacterium_SB2
US3C007
