# Supplementary material for: New isolates refine the ecophysiology of the Roseobacter CHAB-I-5 lineage
Source: ISME Commun. 2025 Apr 18;5(1):ycaf068. doi: 10.1093/ismeco/ycaf068 (PMC12075776; doi:10.1093/ismeco/ycaf068)
Supplement: Supplemental_Tables_Figures_captions_ycaf068(1) [file supplemental_tables_figures_captions_ycaf068(1).docx]

## Supplemental Tables and Figures

**Table S1.** Excel spreadsheet containing genome statistics, computed ANI values, metabolic predictions, AMS1 medium recipe and modifications, growth rates for growth experiments, RPKM values from metagenomic recruitment, and microscopic size calculations. Table S1 is hosted at FigShare (https://doi.org/10.6084/m9.figshare.25898389).

**Figure S1**. Phylogenetic tree of 16S rRNA gene sequences from the Alphaproteobacteria with US3C007 and other CHAB-I-5 representatives. Nodes outside of the CHAB-I-5 and Roseobacter HIMB11 clade have been collapsed to show US3C007’s inclusion with the CHAB-I-5 sequences. The CHAB-I-5 cluster is boxed in red and strain US3C007 is starred.

**Figure S2.** Phylogenomic tree of all CHAB-I-5 genomes prior to dereplication and those of the sister clade containing AG-337-I11 and others. Scale bar indicates changes per position. Filled circles indicate nodes with bootstrap values ≥ 95%.

**Figure S3.** Phylogenomic tree of dereplicated CHAB-I-5 genomes (excepting the dual copies of the SB2 genome), and associated ANI values. Dotted lines indicate the position of the OceanDNA_b28631 genome, which was removed due to the low ANI values and the long unsupported branch on the tree. Scale bar indicates changes per position. Filled circles indicate nodes with bootstrap values ≥ 95%.

**Figure S4.** Metagenomic recruitment (normalized as RPKM) to all genomes by latitude with non-linear regression lines featuring shading that represents the 95% confidence intervals. The histogram below the RPKM plots shows the sample distribution according to latitude.

**Figure S5.** Metagenomic recruitment (normalized as RPKM) to all genomes by salinity with non-linear regression lines featuring shading that represents the 95% confidence intervals. The histogram below the RPKM plots shows the sample distribution according to salinity.

**Figure S6.** Metagenomic recruitment (normalized as RPKM) to all genomes by temperature with non-linear regression lines featuring shading that represents the 95% confidence intervals. The histogram below the RPKM plots shows the sample distribution according to temperature.

**Figure S7.** Metagenomic recruitment (normalized as RPKM) for the top 5 recruiting genomes according to **A)** latitude, **B)** salinity, and **C)** temperature with non-linear regression lines featuring shading that represents the 95% confidence intervals. Histograms below the RPKM plots show the sample distribution according to the same x-axis variable. Note that while all metagenomic samples had latitude values, the metadata did not always include salinity or temperature, and thus the total number of points in B) and C) are different.

**Figure S8.** Growth curves of strains US3C007 and FZCC0083 for the temperature and salinity experiments. Y-axes are cell concentrations in cells/ml, x-axes are time. Conditions are written at the top of each plot.

**Figure S9.** Notes and marks for the analyses of cell morphologies. Using the pixel and scale features in Concepts for iPad v6.13, we measured the radii (R) and area of the cross section (S) of the cells. The formula of the lengths (l), volumes (V), and surface areas (SA) calculated based on r (we denoted r as the mean radii of each cell) and S are shown at the top of the figure. The detailed formula could also be found at **Table S1**.

**Figure S10.** Same as Figure S9, marks of measurements for the SEM images.

**Figure S11.** Relative abundance of Subclusters 1 (green) and 2 (blue) compared to temperature. Subcluster RPKMs were summed as in Figure 2B. R^2^ values for the linear regressions are plotted at the top. Shading around the linear regression indicates 95% confidence intervals.

**Figure S12.** Comparison of coverage vs. abundance for the five top-recruiting genomes using multiple approaches. A) Coverage calculated by SAMtools after read recruitment and RPKM calculation via RRAP for samples from Pearl River and San Francisco Bay (SFB). B) Mean trimmed coverage vs. covered genome fraction for Chesapeake Bay samples using CoverM.
